# The Rosencrantz Coin: Predictability and Structure in Non-Ergodic Dynamics—From Recurrence Times to Temporal Horizons

**DOI:** 10.3390/e27020147

**Published:** 2025-02-01

**Authors:** Dimitri Volchenkov

**Affiliations:** Department of Mathematics and Statistics, Texas Tech University, 1108 Memorial Circle, Lubbock, TX 79409, USA; dimitri.volchenkov@ttu.edu

**Keywords:** entropy decomposition, characteristic times, non-ergodic dynamics

## Abstract

We examine the Rosencrantz coin that can “stick” in states for extended periods. Non-ergodic dynamics is highlighted by logarithmically growing block lengths in sequences. Traditional entropy decomposition into predictable and unpredictable components fails due to the absence of stationary distributions. Instead, sequence structure is characterized by block probabilities and Stirling numbers of the second kind, peaking at block size n/logn. For large *n*, combinatorial growth dominates probability decay, creating a deterministic-like structure. This approach shifts the focus from predicting states to predicting temporal horizons, providing insights into systems beyond traditional equilibrium frameworks.

## 1. Introduction

Predicting the future state of a system is a fundamental challenge across various fields, from physics and information theory to economics and cognitive science [1]. Simple systems, such as a biased coin, can encapsulate profound insights into uncertainty, entropy, and information dynamics. In traditional ergodic systems, where the dynamics ensure that time averages converge to ensemble averages, predictability is often assessed using entropy and its decomposition into predictable and unpredictable components [2]. These components are characterized by measures such as recurrence time, residence time, and repetition time, which capture the structure of state sequences and quantify the degree of uncertainty (see Section 2).

However, many real-world systems exhibit non-ergodic behavior, where the assumption of visiting all possible states uniformly over time breaks down. In such systems, conventional entropy-based methods become inadequate because there is no stationary distribution to describe the long-term behavior. Instead, non-ergodic systems are marked by prolonged persistence in certain states, leading to sequences dominated by blocks of repeated outcomes. A striking example of such a system can be found in Tom Stoppard’s play *Rosencrantz and Guildenstern Are Dead*, where Rosencrantz experiences an improbable streak of 92 consecutive heads [3] that challenge traditional probabilistic frameworks. This anomaly prompts deeper exploration into non-ergodic dynamics, where long-term correlations lead to prolonged persistence in specific states. The Rosencrantz coin model (Section 3) provides a useful abstraction for studying systems with long-term correlations and memory effects. In this model, the barrier to switching states may remain constant or change intermittently, governed by a stochastic threshold. This behavior results in block-like structures within the sequences, where the lengths of these blocks increase logarithmically over time, leading to sequences dominated by blocks of persistent states. The variance bounds derived in [4] establish limits on the uncertainty of entropy estimates, particularly under multinomial assumptions. The estimation of errors in mutual information and entropy, as detailed by [5], highlights the importance of accounting for finite sample effects when interpreting dynamical systems. In the Rosencrantz coin model, similar principles can be applied to assess the variability of residence times and quantify the robustness of power-law distributions observed in correlated regimes. The model offers insights into the interplay between randomness and determinism, highlighting how predictable patterns emerge even in fundamentally stochastic processes (see Section 5).

To analyze these patterns, we explore the combinatorial structures governing the emerging block sequences, particularly focusing on the Stirling numbers of the second kind, which count the ways to partition sequences into non-empty blocks. The Stirling numbers reveal that for sufficiently long sequences, the most probable partitioning involves blocks of length approximately lnn, where *n* is the sequence length. This combinatorial insight compensates for the lack of a stationary distribution and enables meaningful predictions of the system’s temporal behavior (Section 5).

Our analysis shows that in a non-ergodic system, the balance between combinatorial growth and the exponential decay of block probabilities results in a deterministic-like structure within a fundamentally random process. Instead of predicting individual outcomes, the focus shifts to predicting the length of sequences based on the observed block size. The concept of a *temporal horizon*—the characteristic length of time over which predictable patterns emerge—becomes central in non-ergodic systems. Instead of predicting the next state, the focus shifts to predicting the duration of structured behavior based on observed block sizes (Section 5). The logarithmic utility of time for prediction the future, which reflects diminishing “returns” on prediction, further connects these ideas to hyperbolic time discounting models often found in human and animal decision-making (Section 6). This novel approach offers a deeper understanding of the dynamics of non-ergodic systems, bridging concepts from information theory, combinatorics, and stochastic processes. This approach also builds upon foundational methods in non-parametric entropy estimation as described by Harris [6], who demonstrated how entropy can be reliably estimated even in systems with vast or poorly defined state spaces.

The paper is organized as follows: Section 2 discusses entropy decomposition in stationary Markov chains, highlighting predictable and unpredictable components and introducing the notions of characteristic times. Section 3 introduces the Rosencrantz coin model and explores its stochastic dynamics. In this study, we also explore how the logarithmic asymptotic integral of motion governs the residence times in the Rosencrantz coin model. By maximizing entropy under a fixed logarithmic mean constraint, we demonstrate that residence time distributions transition from exponential to power-law behavior, emphasizing the dominance of rare, extended events and their connection to scale-invariant dynamics (see Section 4). Section 5 delves into the structure and dynamics of sequences in the non-ergodic Rosencrantz coin model, focusing on the combinatorial properties of block partitions. Section 6 presents a detailed discussion of the findings, and Section 7 concludes the paper by summarizing the key insights and implications for non-ergodic systems.

## 2. Decomposition of Entropy in Stationary Markov Chains—Predictable and Unpredictable Information

An outcome of each coin flip, governed by the transition matrix $\left[\begin{array}{cc} p & 1-p\\ 1-p & p \end{array}\right],$ encapsulates a single bit of information. The probability of state repetition, 0≤p≤1 determines how this information is divided into predictable and unpredictable components [2]. When p=1, the sequence becomes perfectly predictable, locking outcomes into stationary patterns like H,H,… (*heads*) or T,T,… (*tails*). Similarly, when p=0, the sequence alternates deterministically: H,T,H,T,…. In contrast, p=12 corresponds to complete randomness, making future outcomes wholly unpredictable.

For long sequences (n≫1) generated by an *N*-state, irreducible, and recurrent Markov chain ${\left\{X_{t}\right\}}_{t\geq 0}$ with a stationary transition matrix $T_{ks}=\mathrm{Pr}\left[X_{t}=s|X_{t-1}=k\right]$, the relative frequency $n_{k}/n$ of visits to state *k* converges to the stationary distribution $\pi_{k}$, such that $\sum_{k=1}^{N}\pi_{k}T_{ks}=\pi_{s}$ for all *s*. The asymptotic constraints $n_{i}/n\to \pi_{i}$ can be interpreted as the asymptotic *integrals of motion*, acting as conserved quantities in the long-sequence limit. The probability of each specific sequence $\left\{i_{1},i_{2},\ldots ,i_{n}\right\}$ becomes extremely small for large n, decreasing as $\pi_{i_{1}}^{n\pi_{i_{1}}}\pi_{i_{2}}^{n\pi_{i_{2}}}\ldots \pi_{i_{n}}^{n\pi_{i_{n}}}$. Consequently, the *fraction* of distinct sequences consistent with the stationary distribution π decreases exponentially with *n*, and their growth rate is given by

(1) $$N_{\pi }\left(n\right)=\frac{n!}{\left(n\pi_{1}\right)!\ldots \left(n\pi_{N}\right)!}≃e^{-n\left[\sum_{k=1}^{N}\pi_{k}\ln\left(n\pi_{k}\right)-\ln n\right]}=\exp\left(-nH_{\pi }\right),\;\;H_{\pi }\equiv -\sum_{k=1}^{N}\pi_{k}\ln\pi_{k}.$$

Here, $H_{\pi }$ is the entropy of the stationary distribution π, characterizing the exponential decay rate of the fraction of sequences constrained by the *N* asymptotic integrals of motion, $n_{i}/n\to \pi_{i}$, as n→∞. While the total number of sequences grows *factorially* with *n*, the proportion of those consistent with the stationary distribution *decreases exponentially* as *n* increases. In their analysis of entropy estimators, ref. [7] demonstrate that, under a regular Markov process, the plug-in estimators converge asymptotically to a normal distribution. This convergence is governed by the spectral properties of the transition matrix and initial conditions. Up to a factor of 1/lnN, the decay rate $H_{\pi }$, corresponds to the Boltzmann–Gibbs–Shannon entropy quantifying the uncertainty of a Markov chain’s state in equilibrium:

(2) $$H\left(X_{t}\right)=-\sum_{k=1}^{N}\pi_{k}\log_{N}\pi_{k}=\sum_{k=1}^{N}\frac{\log_{N}R_{k}}{R_{k}},\;\;R_{k}\equiv \frac{1}{\pi_{k}}=\lim_{n\to \infty }\frac{n}{n_{k}}.$$

The inverse frequency $R_{k}$ in (2) is the expected *recurrence time* of sequence returns to state k. Thus, $\ln R_{k}$ can be termed the *utility function* of recurrence time, quantifying the reduction in diversity of state sequences caused by the repetition of state *k* in the most likely sequence patterns corresponding to the stationary distribution π.

The entropy (2) serves as a foundation for decomposing the system’s total uncertainty into predictable and unpredictable components [2,8,9]. To analyze the information dynamics, we add and subtract the following conditional entropy quantities to $H\left(X_{t}\right)$, grouping the resulting terms into distinct informational quantities:

(3) $$\begin{array}{cc} H\left(X_{t}\right) & =H\left(X_{t}\right)\pm H\left(X_{t+1}|X_{t}\right)\pm H\left(\left.X_{t}\right|X_{t-1}\right)\pm H\left(\left.X_{t+1}\right|X_{t-1}\right)\\ \; & =\overset{E_{\mathrm{ex}}\left(X_{t}\right)}{\overset{︷}{\left(H\left(X_{t}\right)-H\left(X_{t+1}|X_{t}\right)\right)}}+\overset{I\left(X_{t+1},X_{t}|X_{t-1}\right)}{\overset{︷}{\left(H\left(\left.X_{t+1}\right|X_{t-1}\right)-H\left(\left.X_{t}\right|X_{t-1}\right)\right)}}\\ \; & +\overset{H\left(X_{t}|X_{t+1},X_{t-1}\right)}{\overset{︷}{\left(H\left(\left.X_{t+1}\right|X_{t}\right)+\left.H\left(\left.X_{t}\right|X_{t-1}\right)-H\left(\left.X_{t+1}\right|X_{t-1}\right)\right.\right)}}. \end{array}$$

The second term in the above decomposition, $H\left(X_{t+1}|X_{t-1}\right)-H\left(X_{t}|X_{t-1}\right),$ can be naturally identified with the conditional mutual information,

(4) $$I\left(X_{t+1},X_{t}|X_{t-1}\right)=H\left(X_{t}|X_{t-1}\right)-H\left(X_{t}|X_{t+1},X_{t-1}\right).$$

Rewriting $H\left(X_{t}|X_{t-1}\right)$ as

(5) $$H\left(X_{t}|X_{t-1}\right)=H\left(X_{t}\right)-I\left(X_{t};X_{t-1}\right)$$

and expanding the conditional entropies in (3), we arrive at the following:

(6) $$I\left(X_{t+1},X_{t}|X_{t-1}\right)=H\left(X_{t+1}|X_{t-1}\right)-H\left(X_{t}|X_{t-1}\right),$$

which matches the second term of the decomposition.

The excess entropy $E_{\mathrm{ex}}\left(X_{t}\right)$ quantifies the influence of past states on the present and future states, capturing the system’s structural correlations and predictive potential of the system. The conditional mutual information $I\left(X_{t+1},X_{t}|X_{t-1}\right),$ represents the information shared between the current state $X_{t}$ and the future state $X_{t+1}$, independent of past history. It vanishes for both fully deterministic and completely random (p=12) systems, reflecting the absence of predictive utility in these extremes. The sum of the excess entropy and the conditional mutual information shown in the second line of (3) represents the *predictable information* in the system [2,8]. This component quantifies the portion of the total uncertainty in the future state $X_{t+1}$ that can be resolved using information about the current state $X_{t}$ and the past states within the system’s dynamics. In contrast, the *unpredictable* component, $H\left(X_{t}|X_{t+1},X_{t-1}\right),$ given in the third line of (3), quantifies the intrinsic randomness in the system that remains unresolved even with full knowledge of its history. The entropy decomposition in (3) is closed, meaning it completely partitions the total entropy $H(X_{t})$ into predictable and the unpredictable components. For a fully deterministic system (e.g., p=1 or p=0), the unpredictable component vanishes, $H(X_{t}|X_{t+1},X_{t-1})=0$, making the entire entropy predictable, $H\left(X_{t}\right)=E_{\mathrm{ex}}\left(X_{t}\right)$. Conversely, for a completely random system (p=12), the predictable components disappear. In this case, $H\left(X_{t}\right)=H\left(X_{t}|X_{t+1},X_{t-1}\right),$ as observations of the prior sequence provide no information for predicting future states ($E_{\mathrm{ex}}\left(X_{t}\right)=0$). Similarly, attempts to predict the next state by repeating or alternating the current state fail, as $I(X_{t+1},X_{t}|X_{t-1})=0$.

For an ergodic process, the entropy of the system is invariant under time shifts. Thus, $H\left(X_{t}\right)=H(X_{t+1}=H\left(X_{t-1}\right)$. This property ensures that the decomposition in (3) holds at any time step *t*, making it applicable to a wide class of systems. This closed decomposition partitions the total entropy $H(X_{t})$ into predictable and unpredictable components, providing a comprehensive framework for analyzing information dynamics.

In a finite-state, irreducible Markov chain, the entropy rate quantifies the uncertainty associated with transitions from one state to the next. It is formally given by

(7) $$H\left(X_{t+1}|X_{t}\right)=-\sum_{k=1}^{N}\pi_{k}\sum_{s=1}^{N}T_{ks}\log_{N}T_{ks}=-\sum_{k=1}^{N}\pi_{k}\log_{N}\left(\prod_{s=1}^{N}T_{ks}^{T_{ks}}\right)$$

where the second formulation highlights the connection to the *geometric mean* of transition probabilities. The term $\prod_{s=1}^{N}T_{ks}^{T_{ks}}$ can be interpreted as the geometric mean time-averaged transition probability rate (per step) from the state *k* over an infinitely long observation period:

(8) $$\prod_{s=1}^{N}T_{ks}^{T_{ks}}=\lim_{n\to \infty }\sqrt[{n}]{\prod_{s=1}^{N}{\left(T_{ks}\right)}^{n_{k,s}}}\equiv Q_{k},$$

where $n_{k,s}/n$ is the observed frequency of transitions from *k* to *s* over the most likely sequence of length *n*. The frequency converges to the transition probability $T_{ks}$ as n→∞. The inverse of the geometric mean transition probability rate, $R_{k}\equiv Q_{k}^{-1}$, represents the average *residence time* in state *k*. The excess entropy $E_{\mathrm{ex}}\left(X_{t}\right)$ takes a form similar to the entropy (2):

(9) $$E_{\mathrm{ex}}\left(X_{t}\right)=H\left(X_{t}\right)-H\left(X_{t+1}|X_{t}\right)=\sum_{k=1}^{N}\pi_{k}\left(\log_{N}R_{k}-\log_{N}R_{k}\right)=\sum_{k=1}^{N}\frac{\log_{N}R_{k}/R_{k}}{R_{k}}$$

where the ratio of average recurrence and residence times, $R_{k}/R_{k},$ measures the *transience* of state *k* in a sequence consistent with the stationary distribution π. Low transience implies that typical sequences are more predictable. Conversely, in the case of maximally random sequences where $R_{k}\approx R_{k}$, states are visited regularly, and the excess entropy (9) does not enhance predictability. The formula for excess entropy $E_{\mathrm{ex}}$ remains valid only for non-deterministic processes where all states can be visited and exited with non-zero probability. If the system remains indefinitely in a single state, the excess entropy cannot be meaningfully defined, as it relies on transitions between states to quantify predictability and structural correlations. In such cases, the entropy of the system is zero, as there is no uncertainty in the system’s behavior.

Similarly, the conditional entropy,

(10) $$H\left(\left.X_{t+1}\right|X_{t-1}\right)=-\sum_{k=1}^{N}\pi_{k}\log_{N}\prod_{s=1}^{N}{\left(T^{2}\right)}_{ks}^{{\left(T^{2}\right)}_{ks}}\equiv -\sum_{k=1}^{N}\pi_{k}\log_{N}Q_{k}^{\left(2\right)}$$

quantifies the uncertainty associated with the statistics of *trigrams* involving two transitions. The transition probability rate,

(11) $$Q_{k}^{\left(2\right)}=\lim_{n\to \infty }\sqrt[{n}]{\prod_{s=1}^{N}{\left(T^{2}\right)}_{ks}^{n_{k,•,s}}}=\prod_{s=1}^{N}{\left(T^{2}\right)}_{ks}^{{\left(T^{2}\right)}_{ks}},$$

represents the asymptotic frequency of trigrams starting with state *k*. Here, $n_{k,•,s}/n$ denotes the number of occurrences of trigrams where the first state is *k*, the last state is *s*, and the intermediate state can be any valid state. The “black dot” (•) is used to indicate any possible intermediate state in the trigram. The relative frequency $n_{k,•,s}/n$ converges to the corresponding elements of the squared transition matrix ${\left(T^{2}\right)}_{ks}$, over long, most likely sequences conforming the stationary distribution π. The conditional mutual information,

(12) $$I\left(X_{t+1},X_{t}|X_{t-1}\right)=H\left(\left.X_{t+1}\right|X_{t-1}\right)-H\left(\left.X_{t}\right|X_{t-1}\right)=\sum_{k=1}^{N}\pi_{k}\log_{N}\frac{Q_{k}}{Q_{k}^{\left(2\right)}},$$

measures the amount of information shared between the current state $X_{t}$ and the future state $X_{t+1}$, independently of the historical context provided by $X_{t-1}$. In the context of coin tossing, the mutual information (12) emerges from uncertainty in choosing between alternating the present coin side (p≳0) or repeating the current side (p≲1) when predicting the coin’s future state. The mutual information vanishes for the fair coin (p=12), where transitions are completely random and independent. It also vanishes for a fully deterministic coin (p=1 or p=0), where past states provide no additional information for predicting future behavior.

When the past and present states of the chain are known, the predictable information about the future state can be expressed as follows:

(13) $$P\left(X_{t}\right)=E_{\mathrm{ex}}\left(X_{t}\right)+I\left(X_{t+1},X_{t}|X_{t-1}\right)=-\sum_{k=1}^{N}\pi_{k}\log_{N}\frac{\pi_{k}}{Q_{k}^{2}/Q_{k}^{\left(2\right)}}=\sum_{k=1}^{N}\frac{\log_{N}R_{k}/R_{k}}{R_{k}},\;\;R_{k}\equiv \frac{Q_{k}^{\left(2\right)}}{Q_{k}^{2}},$$

The conditional probability $Q_{k}^{2}/Q_{k}^{\left(2\right)}$ represents the likelihood that, in a two-step transition starting from *k*, both steps remain in *k*. The inverse conditional probability, $R_{k},$ can be interpreted as the average state *repetition time*—the expected time between instances where state *k* appears consecutively twice. Predictable information, therefore, reflects the balance between recurrences (returns to states) and state repetitions (remaining in the same state). Systems with frequent recurrences and repetitions exhibit high predictability, indicating more organized and regular behavior. In contrast, systems with infrequent recurrences and frequent state changes exhibit low predictability, suggesting a more random and chaotic behavior. According to (3), unpredictable information is then defined as follows:

(14) $$U\left(X_{t}\right)=H\left(X_{t}\right)-P\left(X_{t}\right)=\sum_{k=1}^{N}\pi_{k}\left(\log_{N}R_{k}-\log_{N}\frac{R_{k}}{R_{k}}\right)=\sum_{k=1}^{N}\frac{\log_{N}R_{k}}{R_{k}}.$$

The logarithm of the state repetition time $R_{k},$ which can also be interpreted as the *utility* of state repetition, emphasizes the exponential growth of unpredictability when repetition times increase. Conversely, lower values of $\log_{N}R_{k}$ indicate reduced uncertainty and predictable behavior.

**Figure 1:**
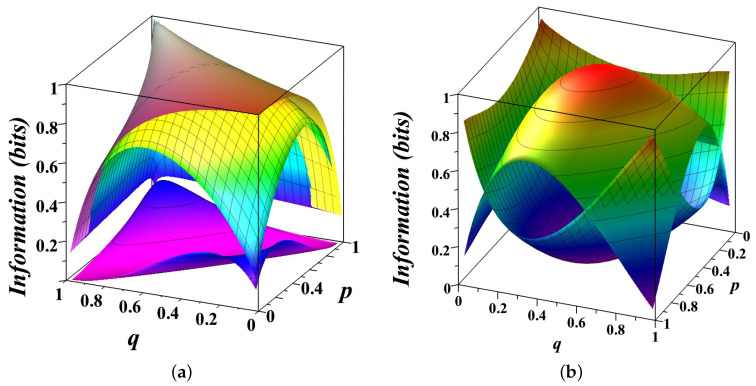
Decomposition of entropy H(p,q) into its components (3) for a biased coin modeled as a Markov chain T(p,q). (**a**) The top surface shows the total entropy H(p,q), the middle surface shows the entropy rate (10), and the bottom surface shows the conditional mutual information (12). The gap between the top and middle surfaces illustrates the excess entropy (9). The gap between the middle and the bottom surfaces corresponds to the unpredictable information component (14) of the system. (**b**) The top, convex surface shows the unpredictable component of information, reaching a maximum of 1 bit for a fair coin (p=q=12), but reducing to zero for a deterministic coin (p,q=0,1). The bottom, concave surface represents the predictable component of the system, which grows as the system becomes more deterministic. Together, these surfaces illustrate the interplay between randomness and structure, showing how predictability increases as *p* and *q* deviate from 12.

Figure 1 provides a detailed visualization of the entropy decomposition (3) for a biased coin modeled as a Markov chain with transition matrix

(15) $$T\left(p,q\right)=\left(\begin{array}{cc} p & 1-p\\ 1-q & q \end{array}\right),$$

where *p* and *q* are the probabilities of repeating the current state.

In Figure 1a, the three surfaces illustrate different information quantities as functions of *p* and *q*. The top surface represents the total entropy $H(X_{t}),$ capturing the overall uncertainty in the system’s state. For a symmetric chain (p=q), the uncertainty reaches its maximum of 1 bit. The entropy decreases when p≠q, as one state becomes more probable than another, reducing overall uncertainty. The middle surface shows the entropy rate $H\left(X_{t+1}|X_{t}\right)$, measuring the uncertainty in predicting the next state given the past states. When p=1−q, this surface coincides with the top one, indicating that the system behaves like a fair coin with no memory of past states. The bottom surface illustrates the conditional mutual information $I\left(X_{t+1},X_{t}|X_{t-1}\right)$, measures the predictive power of the current state for the next state, independent of past history. When p=1−q, the bottom surface highlights the tension between two simple prediction strategies—repeating or alternating the current state—reflecting the inherent randomness of a fair coin. The gaps between these surfaces reveal how the total uncertainty is partitioned. The gap between the top and middle surfaces corresponds to the excess entropy $E_{\mathrm{ex}}\left(X_{t}\right)$, capturing the structured, predictable correlations in the system. Together with the space below the bottom surface, these gaps represent the amount of predictable information $P\left(X_{t}\right)$. The gap between the middle and bottom surfaces represents the unpredictable component of the system, which diminishes as *p* and *q* approach deterministic values (0 or 1), reflecting minimal randomness.

In Figure 1b, the decomposition of entropy is presented more intuitively. The top, convex surface shows the unpredictable component, peaking at 1 bit for a fair coin (p=q=12) and dropping to zero for deterministic scenarios (p,q=0,1). The bottom, concave surface represents the predictable component, which grows as the system becomes more deterministic. Together, these surfaces illustrate the dynamic balance between randomness and structure. The unpredictable component dominates for systems near maximal uncertainty (p=q=12), while the predictable component reflects the emergence of structure as state repetition probabilities deviate from 12.

## 3. Stochastic Dynamics of Rosencrantz’s Coin

In Tom Stoppard’s play “*Rosencrantz and Guildenstern Are Dead*” [3], Rosencrantz improbably wins 92 consecutive coin flips, prompting Guildenstern to speculate that their situation may be influenced by supernatural forces. Indeed, he is correct—for their lives are no longer governed by chance but by the will of the king. Here, we present a simple probabilistic model in which such an improbable sequence can naturally arise, where long-term correlations effectively “freeze” the coin in one of its states. Our model builds on previous approaches using stochastic thresholds to study ecological subsistence dynamics and systems near critical instability points [10].

In this model, each “coin flip” corresponds to a step in a stochastic process. At each step, a random variable *X*, representing the energy or capability to transition, is drawn from a probability distribution Pr{X<x}=F(x). Similarly, the barrier height *Y* is also a random variable with its own distribution Pr{Y<y}=G(y). The process updates *X* at every step, reflecting the inherent randomness in the transition capability. However, the barrier height *Y* updates with a frequency controlled by the parameter 0≤η≤1: with probability η, a new *Y* is drawn from G(y); otherwise, *Y* retains its current value. For η=0, the the barrier is updated at every step, making it maximally volatile. Conversely, when η=1, the barrier height *Y* remains constant, introducing no additional variability. The process remains in the current state at step *t* if $X_{t}\leq Y_{t}$, but transitions to another state if $X_{t}>Y_{t}$. The total number of consecutive successful coin flips, τ, represents the duration of Rosencrantz’s improbable streak—the time the process remains in the state where X≤Y. This framework provides a simple probabilistic explanation for how extraordinary sequences of successes can arise.

The analysis of this model (see [10] for details) depends on the specific distributions F(x) and G(y), as well as on the value of the barrier update probability η. When the barrier is maximally volatile (η=0), *Y* is updated at every step. In this regime, the probability of observing τ successful flips is given by

(16) $$P_{\eta =0}\left(\tau \right)={\left(\int_{0}^{1}dG\left(y\right)dF\left(y\right)\right)}^{\tau }\int_{0}^{1}dG\left(y\right)\left(1-F(y)\right),$$

which decays exponentially with τ, regardless of the specific form of F(x) and G(y). The expected duration of state in such a sequence is always finite,

(17) $$\sum_{\tau =0}^{\infty }\tau \cdot P_{\eta =0}\left(\tau +1\right)<\infty .$$

For uniform distributions dF(u)=dG(u)=du over [0,1], the coin behaves like a fair coin, and the probability of τ consecutive successes simplifies to the following:

(18) $$P_{\eta =0}\left(\tau \right)=2^{-(\tau +1)},$$

and the expected duration of state equals $\sum_{\tau =0}^{\infty }\tau \cdot 2^{-(\tau +1)}=1.$

When η=1, the barrier *Y* remains constant throughout the process. In this case, the probability of τ successful flips is

(19) $$P_{\eta =1}\left(\tau \right)=\int_{0}^{1}dG\left(y\right)F{\left(y\right)}^{\tau }\left(1-F(y)\right).$$

For uniform distributions dF(u)=dG(u)=du over [0,1], the probability (19) reduces to the Beta function $B\left(\tau +1,2\right)=\int_{0}^{1}u^{\tau }\left(1-u\right)du$. Thus, for large τ, the probability of state decays is

(20) $$P_{\eta =1}\left(\tau \right)=B\left(\tau +1,2\right)=\frac{1}{\left(\tau +1)(\tau +2\right)}≃\tau^{-2},$$

The power-law decay in (20) implies that Rosencrantz’s coin appears biased toward producing extended streaks of success, as the expected duration of state diverges, $\sum_{\tau =0}^{\infty }\tau \cdot P_{\eta =1}\left(\tau \right)=\infty$, indicating that blocks of any length can appear in sufficiently long sequences.

For intermediate values of 0<η<1 with uniform distributions dF(u)=dG(u)=du over [0,1], the barrier *Y* alternates between stability and variability. This dynamic creates a mixture of exponential and power-law decay for the probability of τ successful flips [10]:

(21) $$P_{\eta }\left(\tau \right)=\frac{\eta^{\tau }}{\left(\tau +1)(\tau +2\right)}+\sum_{k=1}^{\tau }\frac{\eta^{\tau }}{k(\tau -k+1)(\tau -k+2)}\sum_{m=1}^{k}C_{m,k}{\left(\frac{1-\eta }{\eta }\right)}^{m},$$

where the coefficients $C_{m,k}$ are defined by

(22) $$C_{m,k}=m!\sum_{P(k)}\frac{\prod_{s=1}^{m}l_{s}}{\left(l_{m}+1\right)\prod_{s=1}^{m-1}\left[\left(l_{s}+1\right)\left(k-\sum_{r=1}^{s}l_{r}\right)\right]},$$

and

$$P\left(k\right)=\left\{\left.\{l_{1},\ldots ,l_{m}\}\right|l_{i}\geq 1,l_{1}+\ldots +l_{m}=k,l_{1}\geq l_{2}\geq \ldots \geq l_{m}\right\}$$

represents all integer partitions of *k*.

If $G\left(y\right)=1-{\left(1-y\right)}^{\varepsilon }$, ε>0, the barrier height *Y* is typically close to one, making transitions rare and sharp. When *X* remains uniformly distributed, the probability of τ successful flips follows a Zipf-like behavior [10]:

(23) $$P_{\eta =1}\left(\tau \right)=\frac{\tau^{-1-\varepsilon }}{\zeta (1+\varepsilon )},$$

where $\zeta \left(s\right)=\sum_{n=1}^{\infty }n^{-s}$ is the Riemann Zeta function. This result reflects the dominance of rare, extended streaks of success, evoking a sense of order emerging from randomness. The Rosencrantz coin can also be described by an integral row–stochastic matrix, viz.,

(24) $$T_{\eta_{1},\eta_{2}}\left(t\right)=\left(\begin{array}{cc} P_{\eta_{1}}\left(t\right) & 1-P_{\eta_{1}}\left(t\right)\\ 1-P_{\eta_{2}}\left(t\right) & P_{\eta_{2}}\left(t\right) \end{array}\right),$$

which captures the transition probabilities over a finite interval *t*. Unlike the stationary Markov chain T(p,q) (15), which describes instantaneous transitions, $T_{\eta_{1},\eta_{2}}\left(t\right)$ reflects the cumulative effect of all probabilities up to time *t*.

In the absence of correlations ($\eta_{1,2}=0$), when the height of the energy barrier *Y* changes at every time step and all transitions are statistically independent, the stationary chain T(12,12), corresponding to a fair coin, can be related to the Markov process generator G=1ln2T(12,12)−I for the integral transition matrix $T_{0,0}\left(t\right)$, where *I* is the identity matrix. In this case, the integral matrix $T_{0,0}\left(t\right)=\exp\left(tG\right)$ is the solution of the differential equation ${\dot{T}}_{0,0}\left(t\right)=GT_{0,0},$ with the initial condition $T_{0,0}=I.$ However, when correlated transitions occur ($\eta_{1,2}>0$), the integral matrix $T_{\eta_{1},\eta_{2}}\left(t\right)$ no longer has a straightforward relationship with the instantaneous transition matrix T(p,q).

The long-time behavior of recurrence times in the Rosencrantz coin model depends significantly on the parameter η. The recurrence time $R_{k}\left(t\right)$ is defined as the expected number of steps required for the system to return to state *k* after leaving it. When $\eta_{1,2}=0$, the barrier height *Y* changes dynamically at every step, and the process corresponds to a fair coin. The probability of remaining in a state decays geometrically as $2^{-(t+1)}$. The recurrence time can be calculated as follows:

(25) $$R_{k}=\sum_{t=1}^{\infty }t\cdot P_{\eta =0}\left(t\right),$$

where $P_{\eta =0}\left(t\right)=2^{-(t+1)}$ is the probability of returning to the state after *t* steps. Substituting $P_{\eta =0}\left(t\right)$ into the summation, we find that the recurrence time quickly converges to 2:

(26) $${\left.R_{k}\right|}_{\eta_{1,2}=0}\left(t\right)=2-\frac{2-t}{2^{t}}\to_{t\to \infty }2.$$

This behavior corresponds to a fair coin with a stationary distribution of states π1,2=12. Here, the ergodic hypothesis holds, meaning the system visits all allowable states with equal frequency over a long enough time. Consequently, time averages and ensemble averages become equivalent.

When $\eta_{1,2}=1$, the barrier height *Y* remains fixed, and the recurrence time grows logarithmically. The probability of returning to the state after *t* steps is given by $P_{\eta =1}\left(t\right)=1/t\left(t+1\right),$ which decays more slowly than the geometric decay seen in the η=0. The recurrence time is computed as follows:

(27) $$R_{k}=\sum_{t=1}^{\infty }t\cdot P_{\eta =1}\left(t\right)=\sum_{t=1}^{\infty }\frac{t}{t(t+1)}.$$

This series is related to the digamma function:

(28) $$\Psi \left(x\right)=-\gamma +\sum_{k=1}^{\infty }\left(\frac{1}{k}-\frac{1}{k+x-1}\right),$$

where γ is Euler’s constant. Using this, the recurrence time becomes

(29) $${\left.R_{k}\right|}_{\eta_{1,2}=1}\left(t\right)=2\Psi \left(t+2\right)+\frac{4}{t+2}-4+2\gamma .$$

In this scenario, the ergodic hypothesis does not hold because the recurrence times are not constant. The system’s behavior becomes non-ergodic, meaning it does not visit all states with the same frequency, and ensemble averages lose their equivalence to time averages.

When correlations are present, the Rosencrantz coin shows increasingly prolonged intervals before returning to a given state, rendering the concept of a stationary distribution invalid. The recurrence time grows without settling into a steady pattern, indicating that time averages diverge from ensemble averages and the system’s dynamics are non-ergodic.

## 4. Logarithmic Integral of Motion and Power-Law Distributions of Residence Times

**Figure 2:**
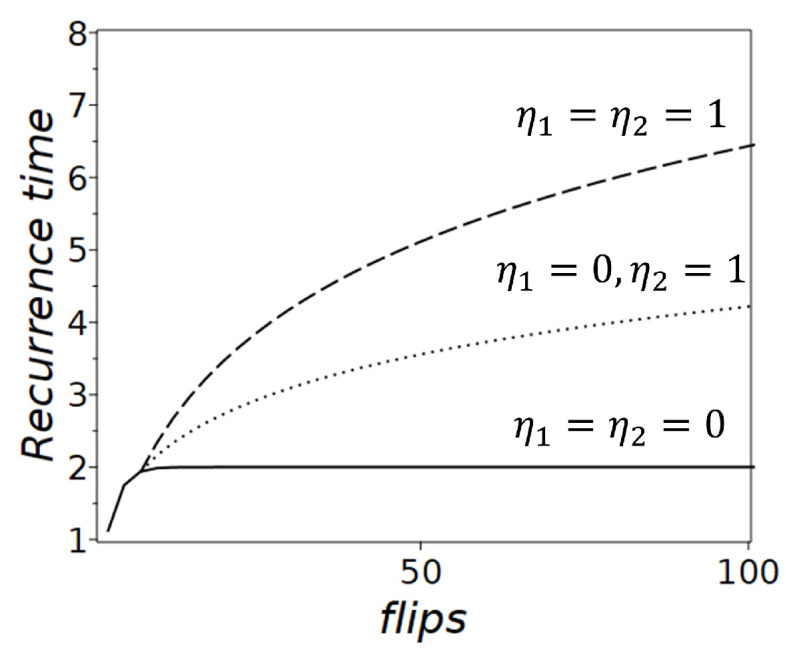
Recurrence times for the Rosencrantz coin model. The curves represent different $\eta_{1}$ and $\eta_{2}$: when $\eta_{1,2}=0$ (solid line), shows a stable recurrence time of 2, consistent with a fair coin and the ergodic hypothesis; $\eta_{1}=0,\;\eta_{2}=1$ (dotted line) shows gradually increasing recurrence times; when $\eta_{1,2}=1$ (dashed line) shows significantly increasing recurrence times.

In stochastic systems such as the Rosencrantz coin model, the residence time—defined as the duration a system remains in a given state before transitioning—is governed by cumulative system dynamics. Importantly, the presence of a logarithmic asymptotic ‘integral of motion’, such as 〈logτ〉, featuring a logarithmic growth of the residence time with increasing steps (Figure 2), shapes the statistical behavior of residence times and their distribution. This growth, rather than the static probabilities of being in a specific state, determines the emergent long-term dynamics and reflects whether the system is ergodic or non-ergodic.

For maximizing entropy $H=-\sum_{\tau }P\left(\tau \right)\log P\left(\tau \right)$ under the fixed logarithmic mean, $\langle \log\tau \rangle =\sum_{\tau }P\left(\tau \right)\log\tau ,$ and the probability normalization condition $\sum_{\tau }P\left(\tau \right)=1$, we define the functional

(30) $$L=-\sum_{\tau }P\left(\tau \right)\log P\left(\tau \right)+\lambda_{1}\left(\sum_{\tau }P\left(\tau \right)-1\right)+\lambda_{2}\left(\sum_{\tau }P\left(\tau \right)\log\tau -\langle \log\tau \rangle \right)$$

where $\lambda_{1,2}$ are the Lagrange multipliers. The variation problem for the entropy maximum under the constraints involving the logarithmic function is considered in [11]. Taking the variation of the functional (30) with respect to P(τ), we obtain the following:

(31) $$\frac{\partial L}{\partial P(\tau )}=-\log P\left(\tau \right)-1+\lambda_{1}+\lambda_{2}\log\tau =0.$$

Solving (31) for P(τ), we obtain $P\left(\tau \right)\propto \tau^{-\lambda_{2}},$ where the exponent $\alpha =\lambda_{2}$ is determined by the value of 〈logτ〉 and the normalization condition $\sum_{\tau }\tau^{-\alpha }=1$. The resulting probability distribution has the power-law form compatible with (20) and (23), viz.,

(32) $$P\left(\tau \right)=\frac{\tau^{-\alpha }}{\zeta (\alpha )},$$

where ζ(α) is the Riemann zeta function ensuring normalization. This distribution reflects the dominance of rare, long residence times for small α, a hallmark of systems exhibiting scale-invariant dynamics.

In systems where 〈logτ〉 is conserved, the logarithmic mean acts as a constraint, favoring configurations where the majority of residence times are short, but a non-negligible fraction exhibits extremely long durations. For the Rosencrantz coin model, a similar principle applies. When the barrier dynamics enforce a logarithmic constraint on residence times, the most likely distribution P(τ) transitions from exponential (η=0) to power-law (η=1) behavior,

(33) $$P\left(\tau \right)∼\tau^{-\alpha },\;\alpha =1+\varepsilon ,$$

where ε>0 reflects the degree of long-range correlations in the system. This framework underscores the emergence of extended streaks of successes or failures as a natural consequence of the interplay between entropy maximization and logarithmic constraints. Our model’s findings on the logarithmic growth of residence times aligns with Harris’s emphasis on logarithmic utility functions for entropy estimation [6]. This shared focus underscores the importance of scaling laws in non-parametric systems, where predictability emerges not from state probabilities but from temporal patterns.

## 5. Structure and Dynamics of Sequences in the Non-Ergodic Rosencrantz Coin Model

To study the sequences generated by the non-ergodic Rosencrantz coin, entropy and its decomposition into predictable and unpredictable components are inapplicable because there is no stationary distribution. Figuratively speaking, the coin “sticks” in each of its states, meaning the structure of observed sequences is characterized by blocks of varying length where the coin remains in the same state for an extended period of time. As we have seen from the analysis of recurrence times (Section 3), the length of such blocks gradually increases, so that increasingly extended streaks of success would be observed in sufficiently long sequences. Because the expected recurrence times grow logarithmically, the system’s dynamics become increasingly non-stationary, and the typical assumptions of equilibrium and steady-state distributions no longer apply. The probability to observe such a sequence, consisting of *m* consecutive blocks of lengths $n_{1},\ldots ,n_{m}$, is characterized by the product $\prod_{i=1}^{m}P_{\eta =1}\left(n_{i}\right)$, where $n=n_{1}+\ldots +n_{m}$ is a partition of *n* into *m* terms.

The number of ways to partition a sequence of *n* elements into *m* blocks is given by the Stirling numbers of the second kind S(n,m) [12], which satisfy the recurrence relation:

(34) S(n+1,m)=S(n,m−1)+mS(n,m).

**Figure 3:**
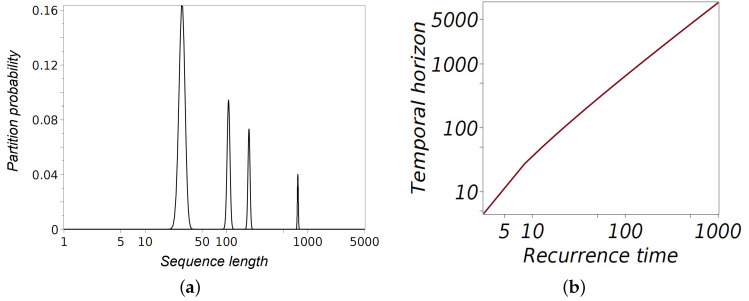
(**a**) Partition probabilities for sequences of lengths 100, 500, 1000, and 5000. The graph displays the Stirling numbers of the second kind, normalized by the corresponding Bell numbers, representing the probability of partitioning a sequence of length *n* into *m* non-empty subsets. The sharp peaks, located at m≃O(n/lnn), indicate the maxima of the Stirling numbers, highlighting the most probable partition configurations. The horizontal axis uses a logarithmic scale to effectively capture the wide range of sequence lengths. (**b**) Temporal horizon n as a function of recurrence time R for a non-ergodic Rosencrantz coin.

The values of S(n,m) are not uniformly distributed across all possible block sizes but exhibit a maximum for specific values of *m*. Figure 3a shows the normalized Stirling numbers of the second kind, representing the probability of partitioning a sequence of length *n* into *m* non-empty blocks. The normalization uses corresponding Bell numbers, $B\left(n\right)=\sum_{1\leq m\leq m}S\left(n,m\right)$, which count the total number of partitions of an *n*-set. This normalization scales large combinatorial values into probabilities, enabling clear graphical representation. The horizontal axis uses a logarithmic scale to depict sequence lengths of 100, 500, 1000, and 5000, while the vertical axis shows the corresponding partition probabilities. The sharp peaks in the distributions highlight the most probable partition configurations for each sequence length.

As *n* increases, certain partition configurations become increasingly dominant due to the combinatorial growth of the Stirling numbers. These configurations correspond to partitions where the block sizes are roughly balanced, maximizing the number of ways the sequence can be divided.

Taking the natural logarithm of S(n,m), we obtain the following:

(35) $$\ln S\left(n,m\right)\approx m\;\ln\left(\frac{n}{m}\right)+O\left(m\right).$$

By differentiating and setting d(mln(n/m))/dm=0, we find the position of the maximum probability to be $m_{\max}=n/\ln n$. A detailed analysis conducted in [13] provides a more accurate estimate:

(36) $$m_{\max}\approx \frac{n}{\ln n}+O\left(\frac{n{\left(\ln\ln n\right)}^{1/2}}{{\left(\ln n\right)}^{3/2}}\right).$$

Substituting this value of $m_{\max}$ back into the asymptotic expression for the logarithm of the maximum Stirling number, we have

(37) $$\ln S_{\max}\approx n\ln n-n-n\ln\ln n+\frac{2n\ln\ln n}{\ln n}.$$

The above estimate suggests that the system’s temporal horizon *n* is most likely partitioned into blocks of size lnn, representing the typical duration spent in a state before switching. The combinatorial advantage that favors specific block sizes reflects the intrinsic structure determined by the Stirling numbers of the second kind for large *n*. The estimates in (37) become increasingly accurate as *n* grows; however, for smaller *n*, correction terms can significantly influence the approximation (see Figure 3).

According to Rennie and Dobson [13], as *n* increases, the most likely partition of the sequence into r=(1±ϵ)n/lnn blocks approaches the configuration described by (37). The deviation between the maximum Stirling number and the actual Stirling number for this partition is given by

(38) $$\ln S_{\max}-\ln S\left(n,r\right)\approx -nH,\;\;H\approx \frac{{\left(\ln\ln n\right)}^{2}}{2\ln n}.$$

The rate of convergence H to the most likely partition structure in (38) can be interpreted as an entropy-like function—a measure of block-related entropy for the non-ergodic system. The value H varies only slightly over a wide range of *n*. Asymptotically, a sequence of length *n* will most likely be divided into $m_{\max}=n/\ln n$ blocks, each of length R≈lnn, which is the characteristic recurrence time for the Rosencrantz coin remaining in a particular state. Consequently, the probability of observing a sequence consistent with the configuration of maximum likelihood equals to

(39) $${\left[P_{\eta =1}\left(\ln n\right)\right]}^{n/\ln n}\approx \exp\left(-\frac{2n\ln\ln n}{\ln n}\right)=\exp\left(-\frac{2n\ln R}{R}\right),$$

being compensated by the last correction term in the asymptotic approximation for the maximum Stirling number (37)—for blocks of the most probable size, the power-law decay over time is offset by the combinatorial growth of partitions. The term 2lnR/R in (39) corresponds to the entropy of a stationary coin, as discussed earlier in (2). It captures the balance between the combinatorial growth of the predominant partitions and the algebraic decay of block probabilities (20), which stabilizes the characteristic block size at lnn. For sufficiently large *n*, the combinatorial increase in the number of predominant partitions compensates for the decay in block probabilities, ultimately dominating the system’s behavior. Consequently, the structure of generated sequences resembles a deterministic schedule rather than a purely random process. The deterministic-like pattern emerges because the recurrence time R aligns with the residence and repetition times, making the sequence’s structure predictable. This conclusion fully aligns with the case of a biased coin T(p,q), where the probabilities *p* and *q* of repeating a state are close to one. Under these conditions, the coin’s behavior becomes almost entirely predictable (see Figure 1).

For such a non-ergodic system, the relevant question is not whether the next state of the coin can be predicted, but whether the *temporal horizon* n—the length of the sequence of flips that can be predicted based on the observed characteristic block size R. Solving the equation R=n/lnn gives the following:

(40) $$n\left(R\right)=-R\;\;{\mathrm{LambertW}}_{-1}\left(-R^{-1}\right),$$

where ${\mathrm{LambertW}}_{-1}\left(x\right)$ refers to the −1-th branch of the Lambert function, defined as the inverse of $ye^{y}=x.$ This branch is real-valued for $-e^{-1}\leq x<0$ and decreases monotonically towards negative infinity as x→0 (R→∞) along the negative real axis, which forms the branch cut [14]. Figure 3b illustrates the relationship between the recurrence time R and the temporal horizon n as given by (40). The curve demonstrates that as the recurrence time, derived from the optimal block size determined by combinatorial analysis, increases, the temporal horizon grows super-linearly. This relationship implies that the longer the system remains in a state before switching, the further into the future one can predict its behavior. Solving R=n/lnn via the Lambert function (40) also provides a quantitative prediction of how long structured behavior will persist.

## 6. Discussion—Temporal Horizons and the Predictive Economy of Non-Ergodic Systems

In the study of non-ergodic systems like the Rosencrantz coin model, the temporal horizon refers to the characteristic duration over which predictable patterns and structures emerge within the system’s evolution. Unlike ergodic systems, where time averages converge to ensemble averages, non-ergodic systems fail to visit all possible states uniformly. Consequently, the system’s long-term behavior cannot be fully captured by stationary probability distributions.

This limitation shifts the analytical focus to understanding the durations between key events, such as state switches or recurrences. In such cases, combinatorial insights compensate for the absence of a stationary distribution, enabling meaningful temporal predictions in systems governed by deterministic-like patterns amidst randomness. The interplay between the recurrence time and the temporal horizon provides a robust framework for predicting the extent of structured behavior in non-ergodic processes.

Partitioning these sequences into blocks of persistent states reveals a deep connection to combinatorial structures, particularly the Stirling numbers of the second kind. These numbers exhibit a sharp maximum for partitions of size lnn. For sufficiently large *n*, the combinatorial growth in the number of partitions compensates for the decay in block probabilities $\prod_{i=1}^{m}P_{\eta =1}\left(n_{i}\right)$ for the most likely partitions $n=n_{1}+\ldots +n_{m}$. This balance between increasing block lengths and decreasing block probabilities results in a deterministic-like structure within an inherently stochastic system.

The temporal horizon, defined by the duration over which predictable patterns emerge, offers a new perspective for analyzing non-ergodic processes. The logarithmic recurrence time R≈lnn can be interpreted as the *prediction utility of time* within a conceptual *prediction economy*. The logarithmic utility of time implies that predictable patterns arise over increasingly longer horizons. Although the first derivative $R^{\prime }\left(n\right)>0$, indicating that additional time enhances prediction, the second derivative $R^{\prime \prime }\left(n\right)<0$ reflects diminishing returns: each subsequent unit of time (or coin flip) adds less predictive value than the previous one. The concave nature of the logarithmic utility highlights this diminishing predictive benefit.

This logarithmic structure seamlessly leads to hyperbolic time discounting in prediction. Hyperbolic discounting prioritizes short-term forecasts with higher certainty over long-term, uncertain ones. The *Arrow–Pratt* measure of risk aversion [15,16] and the *Leland* measure of prudence [17] formalize this idea:

(41) $$-\frac{R^{\prime \prime }\left(n\right)}{R^{\prime }\left(n\right)}=\frac{1}{n},\;-\frac{R^{\prime \prime \prime }\left(n\right)}{R^{\prime \prime }\left(n\right)}=\frac{2}{n}.$$

These measures describe how knowledge of a block’s length improves the ability to predict the temporal horizon. This relationship aligns with the hyperbolic time discounting model, a well-established framework in human [18] and animal [19] intertemporal choice, where immediate rewards are weighted more heavily than future gains. In the Rosencrantz coin model, this perspective underscores the preference for short-term, reliable predictions over long-term, uncertain ones. In his exploration of entropy estimation, Harris [6] identifies significant challenges when probability mass is distributed across numerous small classes. In the Rosencrantz coin model, similar obstacles arise due to the absence of stationary distributions. By employing logarithmic scaling and Stirling numbers of the second kind, we provide a robust framework for analyzing residence times and sequence predictability.

## 7. Conclusions

In this paper, we explored the dynamics of both ergodic and non-ergodic systems through the lens of a coin-flipping model, specifically focusing on the Rosencrantz coin that can “freeze” in one of its states. We compared the behavior of a conventional biased coin, characterized by predictable and unpredictable entropy components, to the non-ergodic Rosencrantz coin, where the absence of a stationary distribution alters the predictability framework.

In the ergodic case, entropy decomposition into predictable and unpredictable components relies on characteristic times such as recurrence, residence, and repetition times. These measures provide insights into the structure and behavior of state sequences generated by stationary Markov chains. However, in the non-ergodic Rosencrantz coin model, where the system can persist in a state for extended periods, traditional entropy decomposition becomes inapplicable. Harris’s seminal work on non-parametric entropy estimation provides critical insights into how entropy can be measured in systems with large or infinite state spaces [6]. While our model does not explicitly rely on sampling from a multinomial population, the estimation challenges addressed in Harris’s framework resonate with the Rosencrantz coin, where the combinatorial structure compensates for the lack of a stationary distribution.

Our analysis demonstrated that the Rosencrantz coin’s non-ergodic dynamics lead to logarithmically increasing block lengths in sequences of persistent states. By leveraging the Stirling numbers of the second kind, we identified the most probable partition size of logn and showed that combinatorial growth in the number of partitions compensates for the algebraic decay of block probabilities. This balance results in deterministic-like structures emerging from fundamentally stochastic processes. Our analysis of the Rosencrantz coin model reveals that the logarithmic asymptotic integral of motion, 〈logn〉, plays a pivotal role in shaping residence time distributions. The transition from exponential to power-law behavior reflects the system’s ability to balance short-term predictability with the emergence of rare, extended events. This finding underscores the broader relevance of logarithmic constraints and entropy maximization in understanding the temporal dynamics of complex systems.

The concept of a temporal horizon n, defined by the recurrence time R, provides a framework for predicting the duration of structured behavior in non-ergodic systems. The logarithmic utility of time implies diminishing predictive returns as the temporal horizon extends, aligning with hyperbolic time discounting models seen in human and animal decision-making.

In conclusion, the Rosencrantz coin model reveals that in non-ergodic systems, predictability is less about individual outcomes and more about understanding the length of sequences governed by persistent state blocks. This study bridges information theory, combinatorics, and stochastic processes, offering a deeper perspective on the dynamics and predictability of systems where conventional equilibrium frameworks fail.

## Data Availability

The original contributions presented in this study are included in the article. Further inquiries can be directed to the corresponding author.
